# Effects of Sn doping on the morphology and properties of Fe-doped In_2_O_3_ epitaxial films

**DOI:** 10.1186/1556-276X-7-661

**Published:** 2012-11-30

**Authors:** Tie Zhou, Lin Wei, Yanru Xie, Qinghao Li, Guoxiang Hu, Yanxue Chen, Shishen Yan, Guolei Liu, Liangmo Mei, Jun Jiao

**Affiliations:** 1 School of Physics and State Key Laboratory of Crystal Materials, Shandong University, Jinan 250100, People's Republic of China; 2 School of Information Science and Engineering, Shandong University, Jinan 250100, People's Republic of China; 3 Physics Department, Portland State University, PO Box 751, Portland, OR 97207, USA

**Keywords:** Magnetic semiconductor, Indium oxide, Morphology, Epitaxial film

## Abstract

(Sn, Fe)-codoped In_2_O_3 _epitaxial films were deposited on (111)-oriented Y-stabilized ZrO_2 _substrates by pulsed laser deposition with constant Fe concentration and different Sn concentrations. The influence of Sn concentration on the crystal structure and properties of Fe-doped In_2_O_3 _ferromagnetic semiconductor films has been investigated systematically. Experimental results indicate that Sn doping can effectively reduce the surface roughness and suppresses breakup of the films into separated islands. At the same time, the optical band gap increases and the electrical properties improve correspondingly. However, although the carrier density increases dramatically with the Sn doping, no obvious change of the ferromagnetism is observed. This is explained by a modified bounded magnetic polaron model.

## Background

In the past two decades, diluted magnetic semiconductors (DMSs) have attracted considerable interests due to their novel physical properties and potential applications in spin-based devices [1]. Many material systems of DMSs, such as ZnO, TiO_2_, SnO_2_, In_2_O_3_, GaAs, and also GeMn [2-5], have been widely studied. Among various kinds of oxide DMSs, transition metal-doped In_2_O_3_ has attracted great attention because of its excellent optical and electric properties, and its room-temperature ferromagnetism has been observed in Fe-, Co-, Ni-, and Cr-doped In_2_O_3 _[6-9]. Among these elements, Fe doping is particularly interesting and has attracted lots of attention because of the high solubility (as high as 20%) of Fe ions into In_2_O_3 _lattice and the high magnetic moment of the Fe^3+ ^ion, which makes Fe-doped In_2_O_3 _a fascinating DMS. Many research works have been conducted on Fe-doped In_2_O_3 _films, and high-temperature ferromagnetism was reported by several groups [10-12]. Spin-polarized carriers were also revealed in this material by the anomalous Hall effect (AHE). These results indicate that Fe-doped In_2_O_3 _may be a promising ferromagnetic semiconductor for future spintronic devices. However, for most device applications, smooth surfaces, high crystalline quality, and controllable optical and electrical properties are necessary. Although epitaxial Fe-doped In_2_O_3 _films with room-temperature ferromagnetism and AHE have been grown and studied extensively by now, most works were focused on studying their physical properties and very little effort has been directed toward the growth of high-quality Fe-doped In_2_O_3 _thin films with controlled surface morphology. In our previous work [13], a very rough surface with square-shaped columnar structures was observed in Fe-doped In_2_O_3 _epitaxial films grown on Y-stabilized ZrO_2 _(YSZ) (100) substrates. Similar rough island-like morphology has been observed in undoped In_2_O_3 _epitaxial films grown on YSZ (100) substrates, which is attributed to the thermodynamically preferred island (Volmer-Weber) growth mode [14]. As we know, In_2_O_3 _is not only used as the host of DMSs, but also is the basis of the most important transparent and conductive materials in industry application. Sn-doped In_2_O_3_, so-called indium tin oxide (ITO), is widely applied in optoelectronic devices due to its high optical transparency in the visible range and high electric conductivity. Many works focused on the growth mechanism of ITO films have been reported, which showed that the surface morphology and properties of In_2_O_3 _films can be effectively improved by Sn doping [15-17]. Similarly, it should be practicable to improve the corresponding properties of Fe-doped In_2_O_3 _ferromagnetic semiconductors by doping appropriate Sn in the same way as ITO. Moreover, the carrier density of Fe-doped In_2_O_3 _films will increase with Sn doping accordingly [18,19]. It is generally accepted that the magnetic coupling in DMS is closely related to the carrier concentration, so the Sn doping is desired to modulate the magnetic property of Fe-doped In_2_O_3 _films also. In this paper, (Sn, Fe)-codoped In_2_O_3 _epitaxial films were deposited on YSZ substrates. The effects of Sn doping on the surface morphology and the optical, electrical, and magnetic properties of Fe-doped In_2_O_3 _ferromagnetic films were studied systematically. An apparent improvement of surface morphology was observed as the Sn concentration increased. At the same time, a corresponding increase of optical band gap and carrier density was found. However, no obvious relation between the carrier density and the ferromagnetism of the films was observed, which was explained by a modified bounded magnetic polaron (BMP) model.

## Methods

### Growth of (Sn, Fe)-codoped In_2_O_3 _epitaxial films

Since the lattice parameter of YSZ (cubic structure, lattice parameter 2*a*_YSZ_ = 10.26 Å) is similar to that of In_2_O_3 _(cubic bixbyite structure, *a*_In2O3 _= 10.118 Å) with the lattice mismatch smaller than 1.6% [13], epitaxial growth of Fe-doped In_2_O_3 _films on YSZ substrates is expected. In this letter, a (111)-oriented single-crystal YSZ substrate was chosen due to the fact that the (111) surface of In_2_O_3 _has the lowest energy amongst the low-index surfaces, which is beneficial to the epitaxial growth. (Sn, Fe)-codoped In_2_O_3_ thin films were deposited by pulsed laser deposition (PLD) at a substrate temperature of 600°C. The stoichiometric targets were prepared from high-purity (99.99%) In_2_O_3_, Fe_2_O_3_, and SnO_2 _powders. For all the targets, the atom ratio Fe/(In + Fe + Sn) is fixed at 5%, and Sn/(In + Fe + Sn) is 0%, 1%, 3%, and 5%, respectively. The powders were mixed in a mechanical ball mill for 5 h, pressed into a 4-cm-diameter pellet, and then sintered at 1,350°C for 10 h in the air. The targets were ablated using a KrF excimer laser (COMPexPro 201, Coherent Inc., Santa Clara, CA, USA) with a pulse repetition rate of 1 Hz and an energy of 400 mJ/pulse for 5,000 pulses, which produces a film with a thickness of about 100 nm. During deposition, the pressure in the PLD chamber was maintained at high vacuum (about 4.0 × 10^−5 ^Pa). After the deposition, the samples were cooled down naturally with the system at the same pressure.

### Characterization of the (Sn, Fe)-codoped epitaxial films

The crystal structure of the (Sn, Fe)-codoped films was analyzed by X-ray diffraction (XRD; XD-3, PG Instruments Ltd., Beijing, China) and high-resolution X-ray diffraction (HRXRD; D8-Discover, Bruker Corp., Karlsruhe, Germany) with Cu K_α _radiation (*λ* = 0.15406 nm). The surface morphology was characterized by atomic force microscopy (AFM; Solver P47 PRO, NT-MDT Co., Moscow, Russia) under contact mode. The optical transmittance was measured using an UV-visible dual-beam spectrophotometer (TU-1900, PG Instruments, Ltd., Beijing, China). The transport properties of the films were determined by Hall effect measurement in the van der Pauw four-point configuration using a SQUID magnetometer (MPMS XL-7, Quantum Design, San Diego, CA, USA). The magnetic measurements were performed using an alternating gradient magnetometer (MicroMag 2900, Princeton Measurements Corp., Princeton, NJ, USA) at room temperature.

## Results and discussion

### Crystal structure and surface morphology

**Figure 1:**
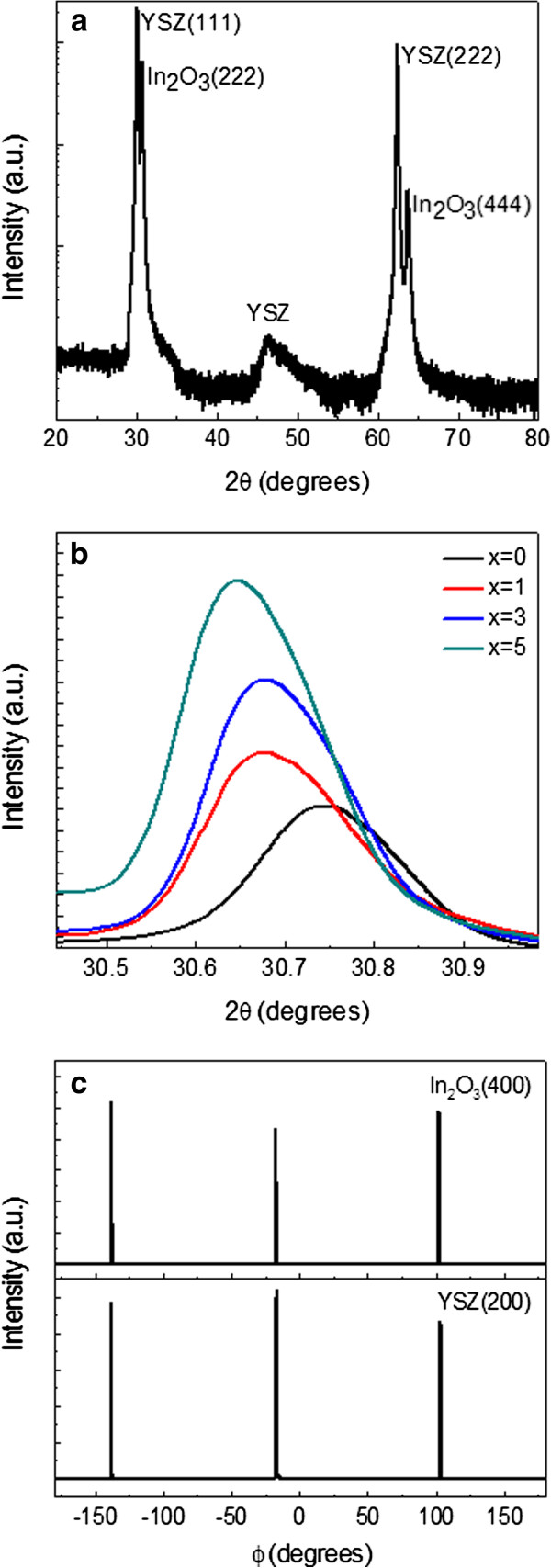
**Crystal structure characterization of the (Sn, Fe)-codoped films.** (**a**) XRD pattern of 5% Sn- and 5% Fe-doped In_2_O_3 _film grown on YSZ (111). (**b**) The expanded views of (222) diffraction peaks of *x*% Sn- and 5% Fe-doped In_2_O_3_ films. (**c**) In-plane rocking curves of (h00) peaks of 5% Sn- and 5% Fe-doped In_2_O_3 _film and YSZ (111) substrate.

A typical X-ray diffraction pattern (*θ*-2*θ* scan) from the 5% Sn- and 5% Fe-doped In_2_O_3 _film grown on YSZ (111) substrate is shown in Figure 1a, and the spectra were plotted on a log scale to better discern any low-level secondary-phase peaks. Sharp peaks corresponding to YSZ (111), (222) and In_2_O_3 _(222), (444) reflections are detected without any other diffraction peaks of In_2_O_3_, suggesting an epitaxial growth of the (Sn, Fe)-codoped In_2_O_3 _film on the (111) surface of the YSZ substrate. No extra peaks relating to the metallic clusters or oxide secondary phases of Fe or Sn are observed in these films within the XRD detection limit, even though the Fe and Sn doping level is as high as 5%. Similar results were obtained from the other samples. Figure 1b shows the expanded views of (222) diffraction peaks of *x*% Sn- and 5% Fe-doped In_2_O_3 _films. As *x* increases from 0 to 1, 3, and 5, the (222) peak shifts from 30.754° to 30.684°, 30.674°, and 30.644°, successively, and the corresponding lattice parameter increases from 10.063 to 10.085, 10.089, and 10.098 Å, respectively. This implies that Sn doping can increase the lattice parameter of Fe-doped In_2_O_3 _[19]. This small increase of the lattice parameter associated with Sn doping leads to improved matching between film and substrate and is beneficial for the epitaxial growth of the In_2_O_3 _film on the YSZ substrate. The out-of-plane rocking scan (not shown here) of the (222) peak is further carried out by HRXRD to check the epitaxial relationship between the film and the YSZ substrate. The full width at half maximum of the peak is 0.194° for the sample without Sn doping and reduces gradually to 0.080° for the sample with 5% Sn concentration. This indicates that Sn doping has a very strong influence on the crystal quality of Fe-doped In_2_O_3 _films. Figure 1c shows the in-plane rocking curves (*Φ* scan) of (h00) peaks of the 5% Sn- and 5% Fe-doped In_2_O_3 _film and YSZ (111) substrate. Threefold symmetry is clear in the in-plane rocking curves. This result further reveals that (Sn, Fe)-codoped In_2_O_3 _films deposited on YSZ substrates are heteroepitaxially grown.

**Figure 2:**
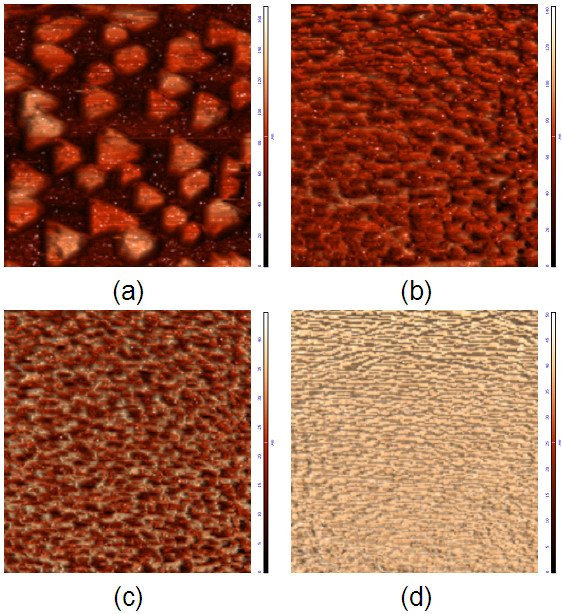
**Morphology of the (Sn, Fe)-codoped films.** AFM images (10 × 10μm^2^) of *x*% Sn- and 5% Fe-doped In_2_O_3 _films grown on YSZ (111) substrates with (**a**) *x* = 0, (**b**) *x* = 1, (**c**) *x* = 3, and (**d**) *x* = 5.

AFM images (10 × 10 μm^2^) of (Sn, Fe)-codoped In_2_O_3 _films are shown in Figure 2. Figure 2a,b,c,d corresponds to Sn concentrations *x* = 0, 1, 3, and 5, respectively. As shown in Figure 2a, the film without Sn doping has a rough surface which is made up of an array of isolated triangle islands with typical edge sizes of about 1 to 2 μm. Electrical transport measurements showed that this sample is highly conductive, which suggests that there is a wetting layer under the islands. Dramatic changes in film morphology caused by Sn doping are observed, as shown in Figure 2b,c,d. The films doped with Sn are much smoother than the undoped film. Continuous films were formed on YSZ (111) substrates without any distinct island structure. As Sn concentration increases, the size of crystal grains decreases gradually, and these crystal grains become more and more smooth and homogeneous. The root mean square surface roughness of the films reduces obviously from 26.029 to 11.609, 5.422, and 4.582 nm as *x* increases from 0 to 1, 3, and 5, respectively, which indicates that Sn doping can smooth the surface of Fe-doped In_2_O_3 _films effectively. The changes in film morphology can be understood considering the increase in lattice parameter of Fe-doped In_2_O_3 _films due to Sn doping, which will lead to better lateral matching with the YSZ substrate, decreased tensile stress in the films, and a smoother surface [17,19]. Furthermore, the Sn dopant may also have a pronounced effect on surface and interface energies which will influence film structure.

### Optical property

**Figure 3:**
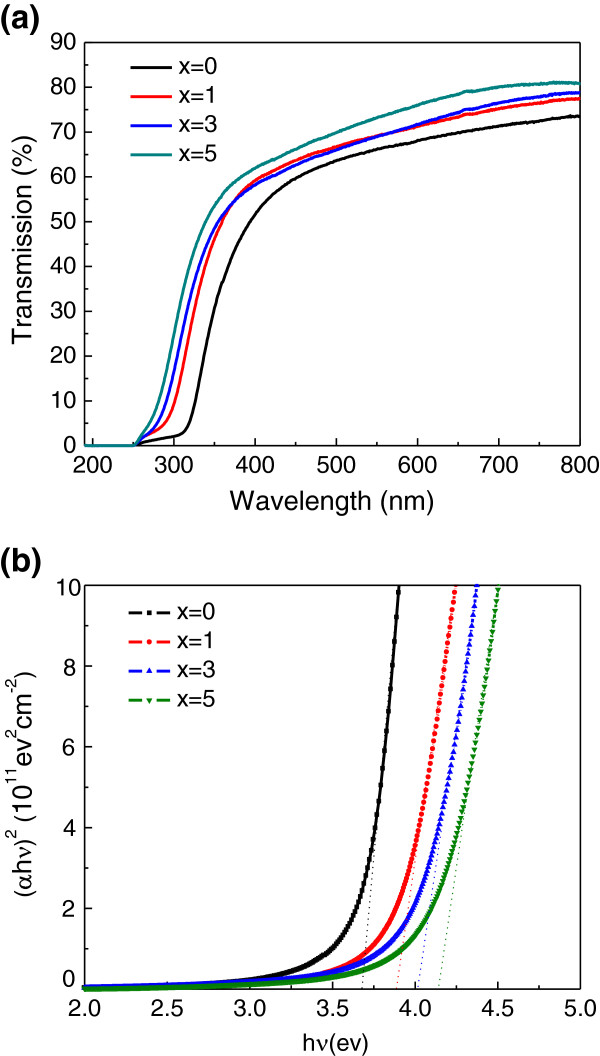
**Optical properties of the (Sn, Fe)-codoped films.** (**a**) Transmittance spectra of *x*% Sn- and 5% Fe-doped In_2_O_3 _films. (**b**) Corresponding plot of (*αhν*)^2 ^versus *hν*. The intercept of linear extrapolation gives the optical band gap.

Figure 3a shows the transmittance spectra of (Sn, Fe)-codoped In_2_O_3 _films. An average transmittance in the visible range is higher than 60% for all samples, and the transmittance in the visible range is enhanced by Sn doping. Moreover, a blueshift in the absorption edge with the increasing doping concentration was also observed, which suggests that the optical band gap of Fe-doped In_2_O_3 _is widened by Sn doping [20,21]. The optical band gap of the films can be calculated from the absorption coefficient and photon energy. The absorption coefficient *α* corresponding to different wavelengths is given by the formula [22]

$$a=\ln\left(\frac{1}{T}\right)/d\text{,}$$

where *T* is the transmittance and *d* is the thickness of the film. The optical band gap *E*_g _can be determined using the relation

$${\left(ah\nu \right)}^{2}=A\left(h\nu -E_{\text{g}}\right)\text{,}$$

where *A* and *hν* are the constant and photon energy, respectively. Figure 3b shows the plot of (*αhν*)^2^ versus *hν* for the films corresponding to those in Figure 3a. The optical band gap *E*_g _is determined by extrapolations of the linear regions of plots to zero absorption (*αhν* = 0). It can be clearly seen that the band gap of (Sn, Fe)-codoped In_2_O_3 _increases from 3.68 to 4.14 eV with the increase of Sn concentration. The widening of the band gap is due to the increase in carrier density as a result of Sn doping. According to the Burstein-Moss model [23,24], the filling up of low-energy states in the conduction band by the doped electrons will widen the band gap [21].

### Electronic transport properties

**Figure 4:**
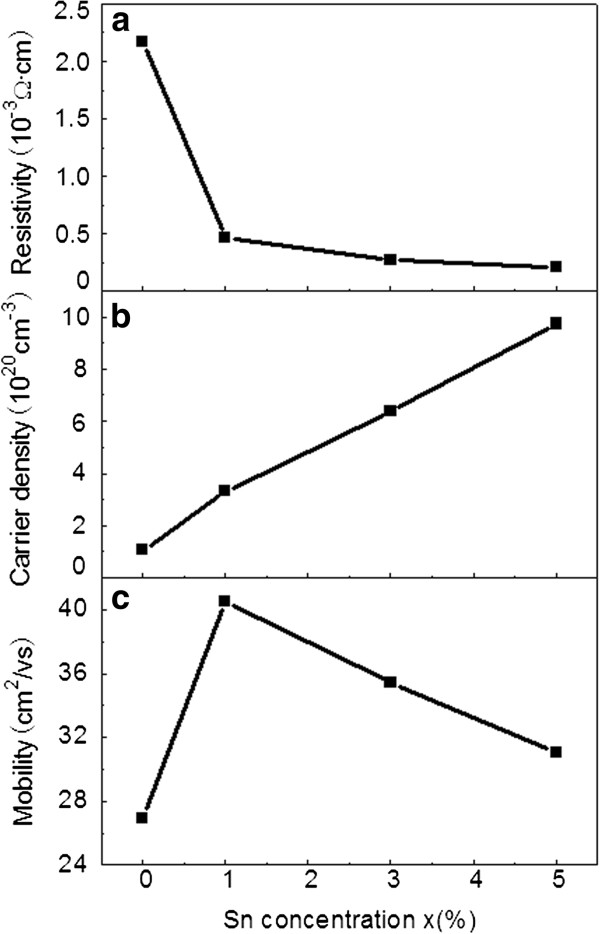
**Transport properties of the (Sn, Fe)-codoped films.** (**a**) Resistivity, (**b**) carrier density, and (**c**) mobility of the (Sn, Fe)-codoped In_2_O_3 _films as a function of Sn concentration.

Figure 4 summarizes the transport properties including resistivity, carrier density, and Hall mobility for (Sn, Fe)-codoped In_2_O_3 _films as a function of Sn concentration. It can be seen that the carrier density increases accordingly with the Sn doping. As Sn concentration increases from 0% to 5%, carrier density increases from 1.065 × 10^20 ^to 9.732 × 10^20 ^cm^−3 ^as a result of the donor electrons from the Sn dopant, while the resistivity decreases by 1 order of magnitude from 2.174 × 10^−3 ^to 2.063 × 10^−4 ^Ω·cm at the same time. A close look at Figure 4a indicates that the initial decrease in resistivity is very sharp with Sn concentration increasing from 0% to 1%, and subsequently, the resistivity tends to saturation with further increase in Sn concentration. This saturation is due to an increase in the density of electron traps introduced by more Sn dopants [18]. On the other hand, the carrier mobility of the films varies nonmonotonously with the Sn concentration (Figure 4c). Initially, the mobility increases from 26.96 to 40.58 cm^2^·V^−1^s^−1 ^as the Sn concentration increases from 0% to 1%. This can be explained by the abovementioned fact that Sn doping decreases the structural disorder and promotes crystalline quality, thereby reduces lattice scattering in the crystal, which increases the mobility [17]. However, as the Sn concentration increases further, the concentration of ionized impurities in the crystal will increase gradually. In that case, ionized impurity scattering is dominant and causes the decrease in carrier mobility [19].

### Magnetic properties

**Figure 5:**
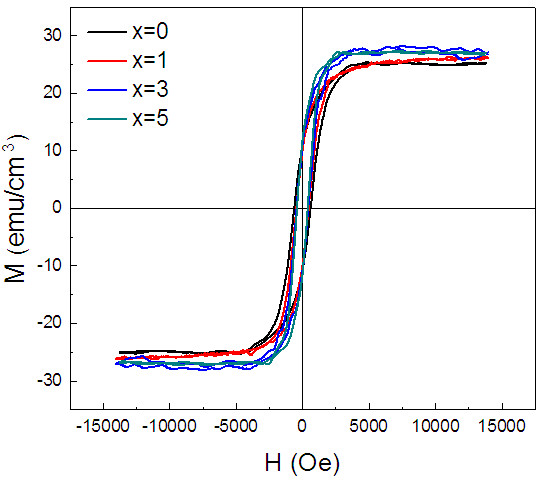
**Hysteresis loops measured at room-temperature of (Sn, Fe)-codoped In**_**2**_**O**_**3 **_**films deposited on YSZ (111) substrate.**

Figure 5 depicts the room-temperature hysteresis loops of (Sn, Fe)-codoped In_2_O_3 _films. The magnetic field was applied in the plane of the film, and the diamagnetic contribution from the substrate has been linearly subtracted from the loops. All the samples display a clear RT ferromagnetic behavior, which is clearly illustrated by the high saturation magnetization and coercive field. Although the carrier density increases with Sn concentration as shown in Figure 4b, the magnetic properties of the films including saturation magnetization and coercivity show no obvious variation with the increase in carrier density. This suggests that the carrier concentration plays an insignificant role in the manifestation of ferromagnetism in Fe-doped In_2_O_3 _films, which cannot be explained by a simple carrier-mediated model.

To explain the ferromagnetism in In_1−*x*_Mn_*x*_As, Ga_1−*x*_Mn_*x*_As, and Ge_1−*x*_Mn_*x *_DMSs, Kaminski and Das Sarma proposed that the spontaneous magnetization arose from a percolation of BMPs [25]. Recently, this model was also used to explain the magnetism in oxide DMSs, such as Cu- and Ag-doped ZnO films [26,27]. In this model, the carriers localized around the oxygen defect strongly couple with the doped magnetic ions and form a BMP sphere. The distance between BMP spheres is determined by the concentration of oxygen defects. When adjacent BMP spheres are sufficiently close to each other, the spin-polarized variable-range hopping between nearby BMP spheres will happen, thus leading to a magnetic coupling between the two BMP spheres. Ferromagnetism phase will form when this sort of BMP coupling percolates throughout the entire film. Within the certain range of defect concentration, the magnetic coupling of BMP spheres increases as the distance between BMP spheres decreases, which is caused by the increase in defect concentration. It was reported that the BMP theory cannot describe the electric property accurately and the concentration of bound polarized carriers derived from the theory is much smaller than the result of the experimental Hall effect measurement [27]. A modified BMP model has been suggested by Chou et al. to interpret and explain both the electric and magnetic properties of the oxide DMS [28,29]. According to the modified BMP model, only carriers in localized states contribute to the magnetic coupling, while other carriers in the conduction band have no discernable effect on ferromagnetism of the samples. In our present case, although Sn doping increases carrier density significantly, the concentration of oxygen defect as the center of the BMP sphere in the films does not change a lot. In addition, the change in the radius of the BMP sphere as a result of the increase in carrier density is so small that it does not vary the number of magnetic irons which are included in the BMP sphere. Consequently, the ferromagnetism of the Fe-doped In_2_O_3 _films does not strikingly change with the increase in carrier density by Sn doping.

## Conclusions

Epitaxial (Sn, Fe)-codoped In_2_O_3 _films with different Sn concentrations were deposited on YSZ (111) substrates by PLD. The crystal structure and surface morphology of Fe-doped In_2_O_3 _films show significant improvement by Sn doping, which is important for future spintronic device application. At the same time, the optical and electric transport properties show disciplinary changes with the Sn concentration. However, contrary to the widely accepted carrier-induced mechanism in oxide DMSs, no significant relation between the ferromagnetism of the films and the carrier density by Sn doping is observed. This result is well consistent with the modified BMP model which suggests that the magnetic coupling in oxide DMSs is mediated by the localized carriers, not the conductive carriers.

## Competing interests

The authors declare that they have no competing interests.

## Authors' contributions

The work presented here was performed in collaboration of all authors. TZ carried out the deposition of the (Sn, Fe)-codoped In_2_O_3 _films and drafted the manuscript. LW conducted the transmittance spectrum measurements. YX carried out the XRD characterization. QL performed the AFM characterization. GH conducted the transport measurement. YC supervised the work and finalized the manuscript. GL helped perform the magnetic measurement. SY and LM analyzed the results and participated in the revision of the manuscript. JJ proofread the manuscript and corrected the English. All authors read and approved the final manuscript.
